# Construction and Comprehensive Analysis of the ceRNA Network to Reveal Key Genes for Benign Tracheal Stenosis

**DOI:** 10.3389/fgene.2022.891741

**Published:** 2022-06-23

**Authors:** Yanpeng He, Chunyan Zou, Zhigang Cai

**Affiliations:** ^1^ The First Department of Pulmonary and Critical Care Medicine, The Second Hospital of Hebei Medical University, Heibei Key Laboratory of Respiratory Critical Care, Shijiazhuang, China; ^2^ Department of Pulmonary and Critical Care Medicine, The First Hospital of Qinhuangdao, Qinhuangdao, China

**Keywords:** benign tracheal stenosis, differential analysis, competing endogenous RNA, PPI, transcriptome sequencing, bioinformatics

## Abstract

**Objective:** To explore the possible biological functions of the differentially expressed genes in patients with benign tracheal stenosis, and to provide a valuable molecular basis for investigating the pathogenesis of benign tracheal stenosis.

**Method:** Whole transcriptome sequencing was performed on blood samples collected from patients with benign tracheal stenosis and normal controls. Differentially expressed mRNA, lncRNA, and circRNA were analyzed using the DESeq2 package. The protein interaction networks for differentially expressed mRNAs were constructed by STRING. The results of gene co-expression network analysis, Starbase database prediction, and differential gene expression were combined to construct a competing endogenous RNA network. The transcription factors of key genes were predicted using the Network Analyst database and a transcription factor-mRNA regulatory network was constructed. The classical pathways, intermolecular interaction networks, and upstream regulatory components of key genes were analyzed using Ingenuity Pathway Analysis (IPA). Finally, the DGIDB database was used to predict the potential therapeutic drugs to target the identified key genes.

**Result:** Based on mRNA, lncRNA and circRNA expression data, we found that differentially expressed mRNAs were enriched in oxygen transport, neutrophil activation, immune response, and oxygen binding. Then the pearson correlation between mRNAs of 46 key genes and lncRNAs and cricRNAs were calculated, and the correlation greater than 0.9 were selected to construct the co-expression network of “mRNA-lncRA” and “mRNA-cricRNA.” Moreover, a “lncRNA-miRNA-mRNA” network and a “circRNA-miRNA-mRNA” network were constructed. IPA analysis showed that the 46 key genes were significantly associated with inflammatory activation and acute respiratory distress syndrome. The constructed TF-mRNA regulatory network was composed of 274 nodes and 573 interacting pairs. 251 potential therapeutic drugs were identified from the DGIDB database.

**Conclusion:** This study analyzed the differential genes associated with benign tracheal stenosis and explored the potential regulatory mechanisms, providing a scientific reference for further studies on the pathogenesis of benign tracheal stenosis.

## Introduction

Benign tracheal stenosis is a common clinical disease in which excessive proliferation of granulation tissue leads to tracheal and bronchial stenosis or complete obstruction due to repeated self-repair after long-term stimulation and injury of tracheal mucosa. The life quality of the patients is seriously affected, which could eventually progress into respiratory failure and death (Sarper et al., 2005). In recent years, with the improvement in surgical technologies and critical care medicine, mechanical ventilation is more often used in clinics, and the incidence of benign tracheal stenosis has increased. Benign tracheal stenosis has many causes, and its pathogenesis mainly results from trauma, injury and inflammation of tracheal mucosa during operation or intubation (Wujtewicz et al., 2009; Bernon et al., 2013). Under these conditions, the fibroblasts begin to proliferate and migrate after induced by inflammatory mediators and growth factors, which increases the production of extracellular matrix and results in the formation of granulation, tissue scar, tissue hyperplasia, and tracheal stenosis (Shitrit et al., 2010; Liang et al., 2015; Kumar et al., 2017; Oberg et al., 2018). However, the molecular mechanism underlying tracheal stenosis has not yet been clarified (Farzanegan et al., 2017). Benign tracheal stenosis is often found after hospitalization, and the delay in diagnosis can affect the treatment efficacy (Shadmehr et al., 2017). Although fiberoptic bronchoscopy can be used for cryotherapy, stent implantation, and other treatments for tracheal stenosis, the recurrence rate and follow-up rate are still high, and there is no better treatment method. The patients with benign airway stenosis had a long survival time and many complications. Therefore, exploring the pathogenic mechanism of tracheal stenosis is of great significance for its early diagnosis and targeted treatment.

The rapid development of gene sequencing technology plays an essential role in the early screening and diagnosis of diseases, as well as the individualized treatment. It greatly improves the speed, efficiency, and sensitivity of detecting circulating free tumor DNA. In addition, high-throughput sequencing technology can accurately detect gene mutations to formulate individualized treatment plan for patients. Also, abnormal genes can be identified through whole genome detection, so that the early intervention treatment can be performed in time (Patch et al., 2015). Through high-throughput sequencing and bioinformatics analysis, it has been reported that about 75% of the human genome can be transcribed into RNA, of which up to 74% are non-protein coding RNA (Kapranov et al., 2007), including long-non-coding RNA (lncRNA), microRNA (miRNA) and circular RNA (circRNA); these RNA species may represent a new disease diagnosis marker and therapeutic target (Beermann et al., 2016; Matsui and Corey, 2017). The regulatory functions of ceRNA provide a new perspective for clarifying the gene expression regulatory network constructed by transcriptome, and add more dimensions for analyzing the molecular mechanism of important biological processes.

In this study, the differentially expressed mRNA, lncRNA and circRNA genes between patients with benign tracheal stenosis and unaffected individuals were explored. And a ceRNA regulatory network was constructed, the main functions and signaling pathways of the key genes were analyzed, and the potential therapeutic drugs were explored. Our results provide a new direction for further investigation on the pathogenesis of benign tracheal stenosis.

## Materials and Methods

### Sample Source and Sequencing

**FIGURE 1:**
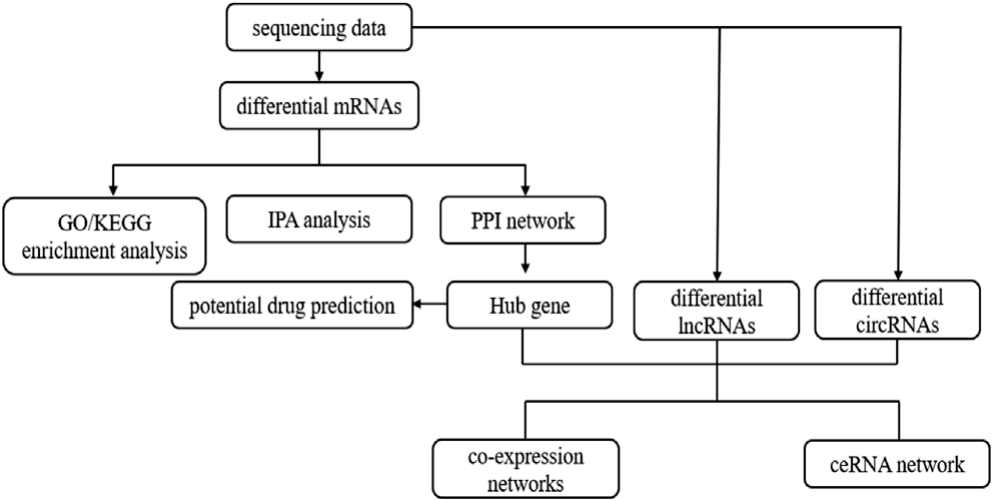
Flowchart describing the schematic overview of the study design.

The benign tracheal stenosis samples and normal control samples were collected in the Second Hospital of Hebei Medical University, including six benign tracheal stenosis samples and five normal control samples. The document number of ethical proof was 2021-R516. This study was approved by the hospital ethics committee and informed consent was obtained from all subjects. The sequencing was completed by Shanghai Shengyin Biotechnology Co., Ltd. Bioinformatics analysis of mRNA-seq datasets was performed according to the workfow illustrated in Figure 1.

### Screening of Differentially Expressed Genes

DESeq2 package (version 1.32.0) was used to screen for differentially expressed mRNA, lncRNA and circRNA between the transcriptomes of diseased and normal samples (Love et al., 2014). The screening conditions were: log2 FC > 1.5, *p* < 0.01. The volcano plot showing the differential expression of genes was plotted using the ggplot2 package of R3.3.5 (Skidmore et al., 2016). The expression heat map of differential genes was plotted using the R pheatmap package (version 1.0.12).

### Enrichment Analysis

The R clusterprofiler package (version 3.18.0) was used to perform enrichment analysis on the differentially expressed genes in gene ontology (go) and Kyoto Encyclopedia of Genes and Genomes (KEGG) (Yu et al., 2012). The enrichment results were visualized using the enrich plot package (version 1.10.2). Go function analysis results included biological process (BP), molecular functions (MF) and cellular components (CC). KEGG pathway analysis was used to understand the involved signaling pathways.

### Construction of Protein-Protein Interaction Network

The protein-protein interaction (PPI) network of differentially expressed mRNA was constructed using the STRING (https://string-db.org) website. The confidence level was 0.4 (Confidence = 0.4). The sparse genes were removed, and the protein network diagram was visualized by Cytoscape software (version 3.8.2) (Shannon et al., 2003). The CytoHubba plug-in in Cytoscape software was used to analyze the interacting genes based on five algorithms [degree (Degree), edge penetration component (EPC), maximum neighborhood component (MNC), maximum neighborhood component density (DMNC), maximum cluster centrality (MCC)], and then the top 100 genes obtained by each algorithm were intersected together to obtain the key genes. Finally, the key genes were enriched and analyzed by Cytoscape plug-in ClueGO.

### Analysis of Co-Expression Network and Construction of Competitive Endogenous RNA Regulatory Network

To analyze the possible interactions between gene products based on the similarity of gene expression, the co-expression network was constructed, which would also help to understand the gene context and find the key genes. As an important hub, key genes play important roles in the network module. First, the Pearson correlation between the key mRNA, lcnRNA, and cricRNA obtained by PPI network was analyzed, and the correlation threshold was set as 0.9. The results were used to construct the co-expression networks of “mRNA-lncRNA” and “mRNA-cricRNA,” which were then optimized by Cytoscap (eversion3.8.2) (Shannon et al., 2003).

The competing endogenous RNA (ceRNA) regulatory networks constructed in this study mainly included circRNA-miRNA-mRNA and lncRNA-miRNA-mRNA. The miRNA associated with key mRNA genes was screened by starbase database (https://starbase.sysu.edu.cn/), and the screening criterion was CLIP-DATA ＞ = 1. In addition, the lncRNA was predicted by miRNA in the starbase database, and the predicted results were intersected with the differentially expressed lncRNA, which yielded the lncRNA-miRNA pairs. The miRNA-mRNA pair was obtained based on the miRNA in the pair. Finally, the two pair groups were matched to construct an “lncRNA-miRNA-mRNA” network. Moreover, the “circRNA-miRNA-mRNA” network and Cytoscape (version 3.8.2) software was used to optimize the results.

### Transcription Factor-mRNA Regulatory Network

In order to visualize the regulatory relationship of gene transcription, NetworkAnalyst 3.0 (https://www.networkanalyst.ca/) was used to predict the transcription factor (TF) of key genes and constructed the TF—mRNA regulation network (Zhou et al., 2019).

### Ingenuity Pathway Analysis

In order to predict the activation or inhibition state of the pathway in which the key genes participated, Ingenuity Pathway Analysis (IPA) on key genes was performed, with Z-score >2 as the significant activation state and Z-score <−2 as the significant inhibition state (Krämer et al., 2014). In addition, the interaction network between molecules was analyzed, and the expression trend between network analysis was observed. Finally, IPA was used to analyze the upstream regulatory elements. The upstream regulator analysis could predict the regulatory transcription factors of key genes and determine whether they may be activated or inhibited; moreover, the action mode of the TFs in this experiment can be predicted by comparing the possible actions of these transcription regulators with the reports from literature.

### Analysis of Key Gene-Drug Interaction Network

In order to explore the potential therapeutic drugs for key gene-related diseases, the targeted drugs of proteins encoded by key genes through the DGIDB database were identified (https://dgidb.genome.wustl.edu/) (Cotto et al., 2018).

## Results

### Differentially Expressed mRNA, lncRNA and circRNA

**FIGURE 2:**
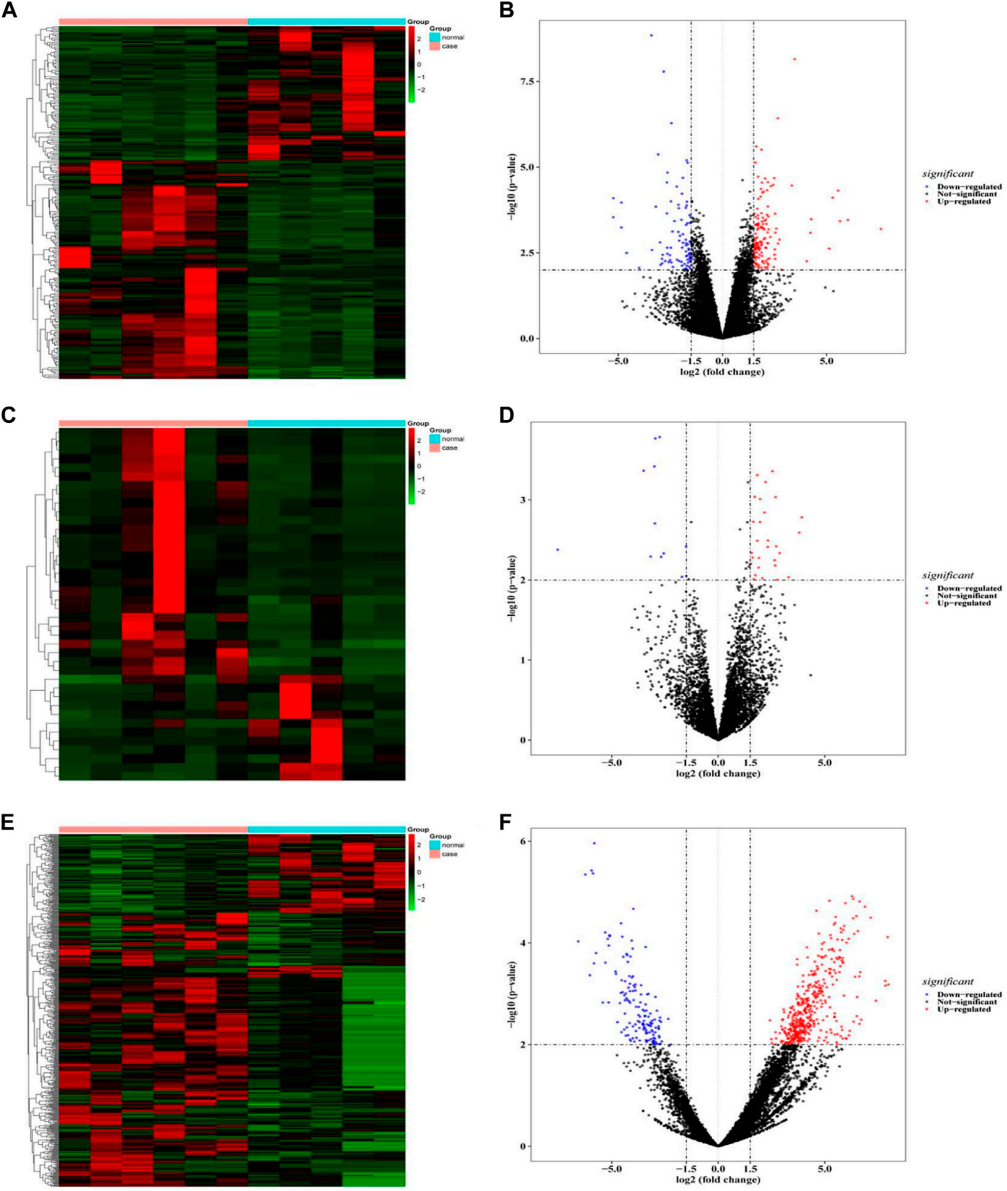
. **(A,B)** mRNA difference analysis heat map and volcano map; **(C,D)** LncRNA difference analysis heat map and volcano map; **(E,F)** CircRNA difference analysis heat map and volcano map.

A total of 229 differentially expressed mRNA were obtained from blood samples of patients with benign airway stenosis and normal controls, of which 146 mRNA were up-regulated and 83 mRNA were downregulated (Figures 2A,B); 40 differentially expressed lncRNA were obtained, of which 28 were up-regulated and 12 were downregulated (Figures 2C,D); 583 differentially expressed circRNA were obtained, of which 434 were upregulated and 149 downregulated (Figures 2E,F). The top5 differentially expressed genes were shown in Supplementary Table S1.

### Enrichment Analysis

**FIGURE 3:**
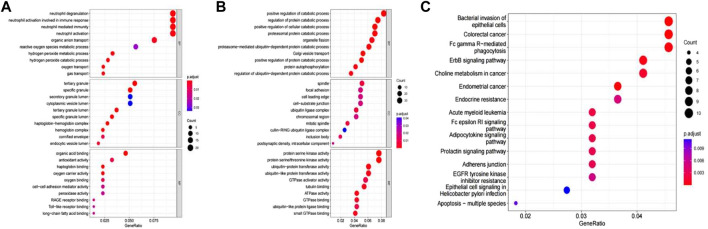
**(A)** Enriched analysis of differentially expressed mRNA in GO; **(B)** Enriched analysis of differentially expressed circRNA in GO; **(C)** KEGG analysis of differentially expressed circRNA.

The differentially expressed mRNAs were mainly enriched in 41 GO processes (*p* < 0.05 and count ≥2), including 14 biological processes, such as neutrophil degranulation, neutrophil activation involved in immune response, neutrophil mediated immunity, neutrophil activation, 11 cellular components, such as tertiary granules, specific granules, and 16 molecular functions, such as organic acid binding, antioxidant activity (Figure 3A). Enrichment analysis of differentially expressed circRNA showed that these circRNAs were related to biological processes such as positive regulation of decomposition process, regulation of protein decomposition process, positive regulation of cell decomposition process, and the involved molecular functions included cell axis, focal adhesion, protein serine kinase activity and protein serine/threonine kinase activity (Figure 3B). The differentially expressed circRNAs were involved in the pathways such as bacterial invasion of epithelial cells, colorectal cancer, FC- γ-mediated phagocytosis, ErbB signaling pathway, and cholinergic metabolism in cancer (Figure 3C).

### Protein-Protein Interaction Construction and Enrichment Analysis of Differentially Expressed mRNA

**FIGURE 4:**
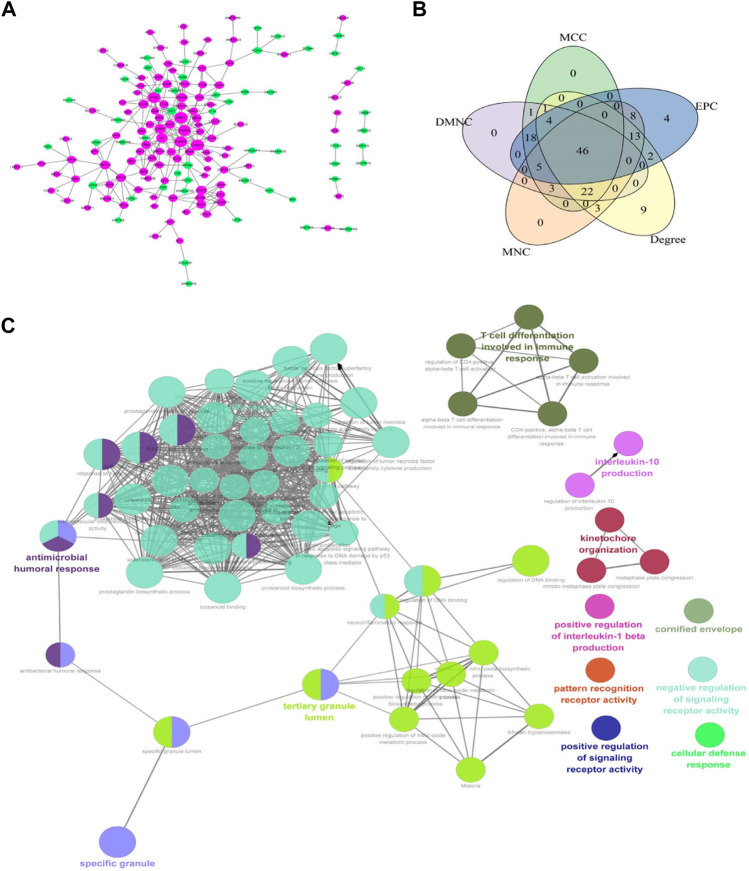
**(A)** Protein interaction network of differential genes. Magenta is an upregulated gene and green is a downregulated gene. The greater the node connectivity, the larger the node; **(B)** Core gene screening; **(C)** ClueGO network of terms/pathways.

The PPI network of 229 differentially expressed mRNA was constructed, and 160 protein interactions were obtained, including 160 nodes and 228 edges (Figure 4A). In the network, MMP9, S100A9, S100A12, S100A8, HP, CAMP, and other upregulated genes were in the center. Five algorithms were used to analyze the interacting genes, and 46 key genes were obtained by intersecting the first 100 genes obtained by each algorithm (Figure 4B), including the calcium binding protein S100A identified in the follow-up PPI network analysis and the hemoglobin A2 (HbA2) gene that was significantly expressed in the mRNA-lncRNA co-expression network. Transcription factor-mRNA regulatory network was the key node of growth and differentiation factor-related serum protein (WFIKKN1) gene and matrix metalloproteinase (MMP) gene. Further GO enrichment analysis of the key genes showed that these genes were mainly related to the regulation of signaling receptor activity, positive regulation of IL-1 *β* production, pattern recognition receptor activity, IL-10 production, T cell differentiation, IL-17 signaling pathway, etc. (Figure 4C).

### ceRNA Network Construction

**FIGURE 5:**
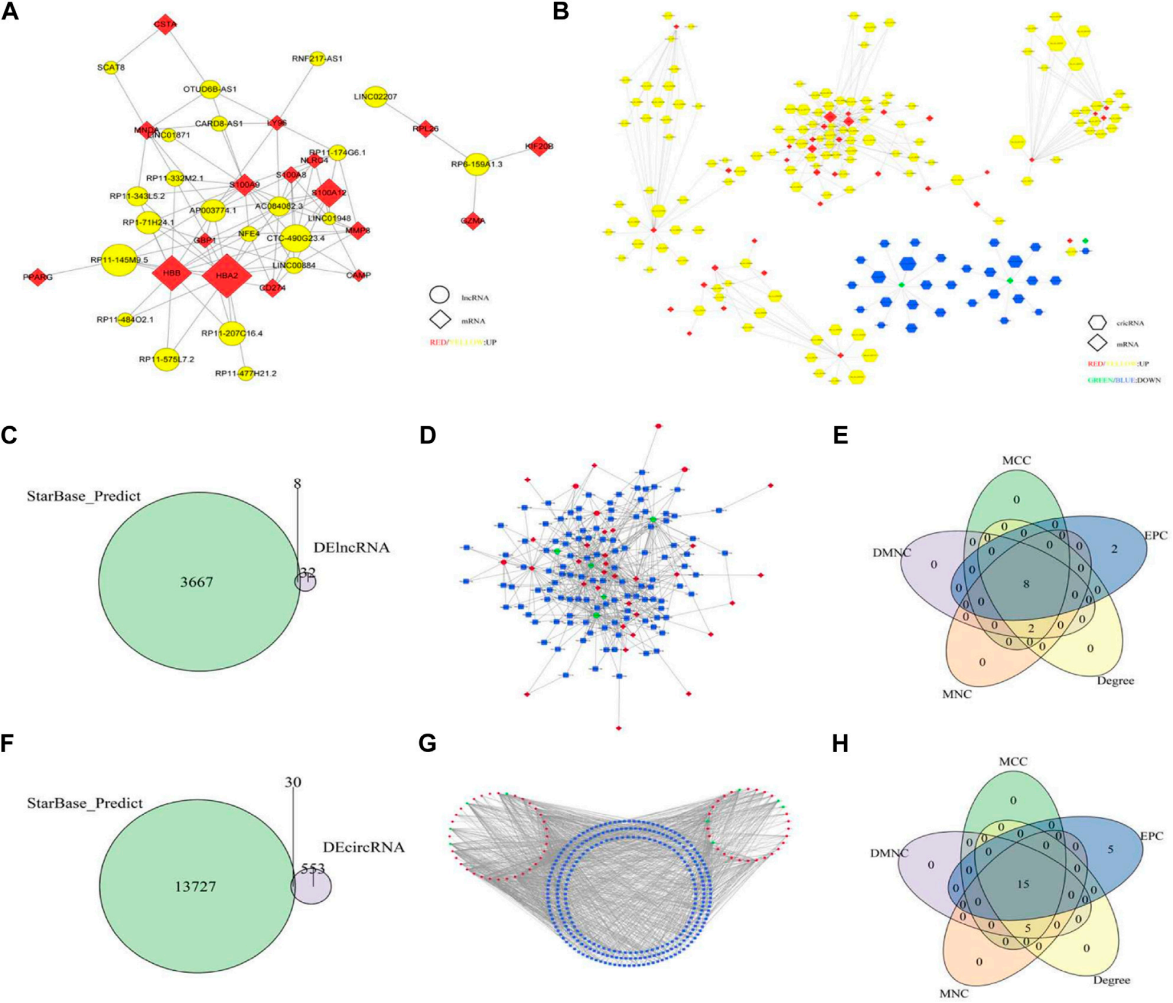
**(A)** mRNA-lncRNA Co-expression network. The diamond is mRNA and the circle is lncRNA. Red and yellow indicate upregulation of mRNA and lncRNA, respectively, and the node size represents the multiple of change in the number of gene markers; **(B)** mRNA-circRNA co-expression network. The diamond is mRNA and the hexagon is circRNA. Red and yellow indicate upregulation of mRNA and up-regulation of circRNA, green and blue indicate downregulation of mRNA and circRNA, respectively, and node size represents multiple changes in gene expression; **(C)** Predicting the intersection of lncRNA and differential lncRNA; **(D)** lncRNA-miRNA-mRNA ceRNA Network; **(E)** The top10 hub gene; **(F)** Predicting the intersection of circRNA and differential circRNA; **(G)** circRNA-miRNA-mRNA ceRNA Network, **(H)** The top20 hub gene.

The gene co-expression network of 46 key genes was constructed to analyze the interaction between genes. LncRNA and cricRNA with more than 0.9 correlation with the 46 key genes were selected, and the co-expression networks of “mRNA-lncRA” and “mRNA-cricRNA” were constructed (Figures 5A,B). The topological features of nodes in all of the networks were shown in Supplementary Table S2.

Through the intersection of lncRNA and differentially expressed lncRNA predicted by starbase database, 8 lncRNA and 128 lncRNA-miRNA pairs were obtained (Figure 5C). Then, according to the 116 miRNA in the lncRNA-miRNA pairs, 31 mRNA and 392 miRNA-mRNA pairs were obtained. The two pair groups were matched to yield a ceRNA network “lncRNA-miRNA-mRNA,” which consisted of 8 lncRNA, 31 mRNA, and 116 miRNA. This network had 155 nodes and 520 relation pairs (Figure 5D). The ceRNA network showed that AP003774.1 might regulate SMC4 through hsa-miR-302d-3p, and LINC01579 might regulate GBP1 through hsa-miR-23a-3p. According to the node score, a total of 10 key genes were screened out (Figure 5E).

Through the intersection with circRNA predicted by starbase data set and differentially expressed circRNA, 30 circRNA and 484 circRNA-miRNA pairs were obtained (Figure 5F). Then, according to the 275 miRNAs in circRNA-miRNA relation pairs, 36 mRNA and 887 miRNA-mRNA pairs were obtained. Finally, the two pair groups were matched and the ceRNA network “circRNA-miRNA-mRNA” was constructed, which had 341 nodes and 1,371 relation pairs (Figure 5G). Moreover, hsa_circ_0000618 might affect the expression of ANXA1 and CD274, and hsa_circ_0001246 might regulate the expression of ANXA1and PIBF1.20 key genes were obtained according to the score of nodes (Figure 5H).

### TF-mRNA Regulatory Network

**FIGURE 6:**
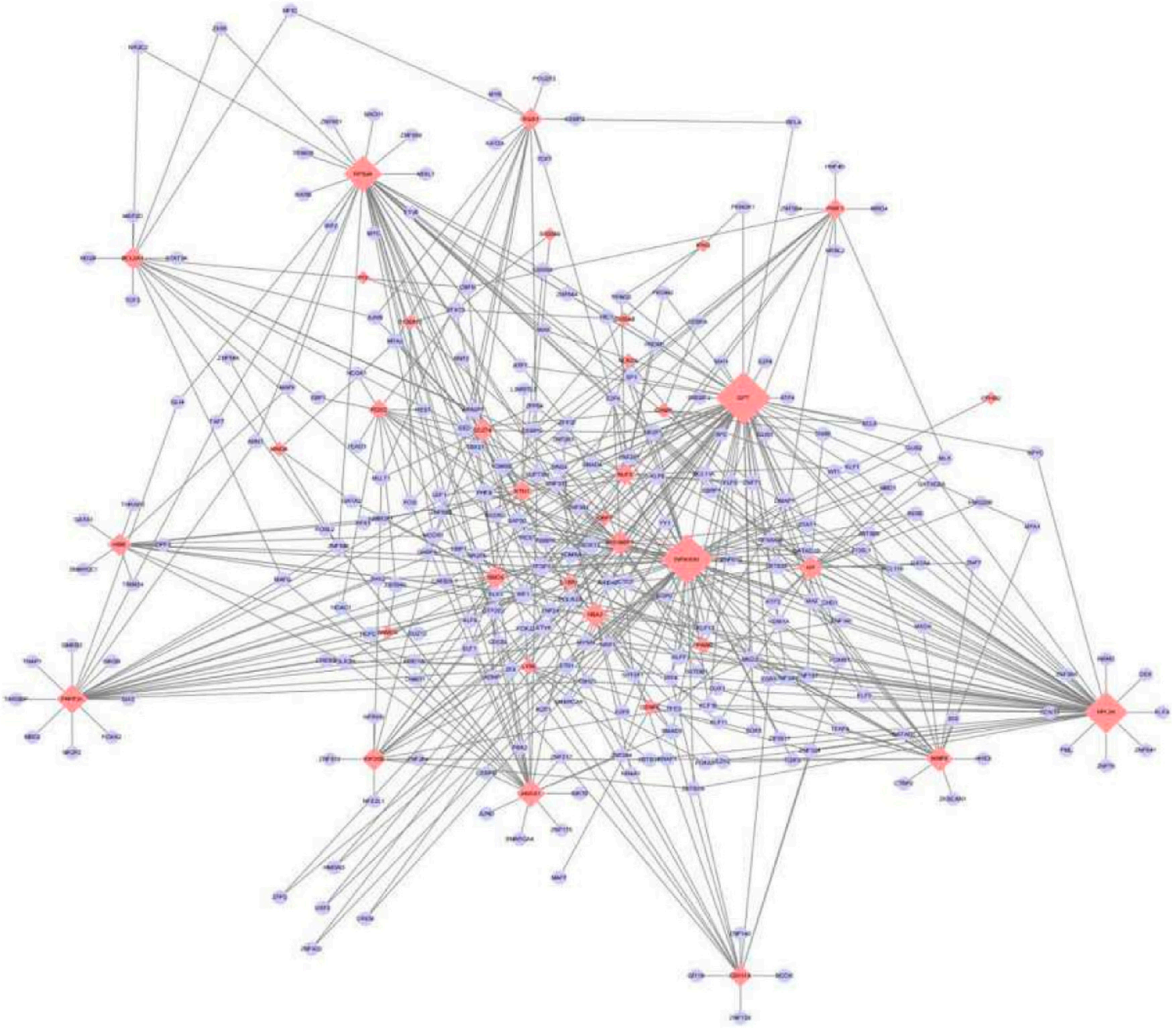
TF-mRNA Regulatory network. The blue oval represents the transcription factor, and the red diamond represents the gene. The greater the degree of connection, the larger the node, the more important it is in the network.

The transcription factors of 46 key genes were predicted, and the TF-mRNA regulatory network was constructed. The network had 274 nodes and 573 edges (Figure 6), in which GPT, WFIKKN1, RPL26 and RPS24 genes were associated with multiple transcription factors.

### Ingenuity Pathway Analysis of Key Genes

**FIGURE 7:**
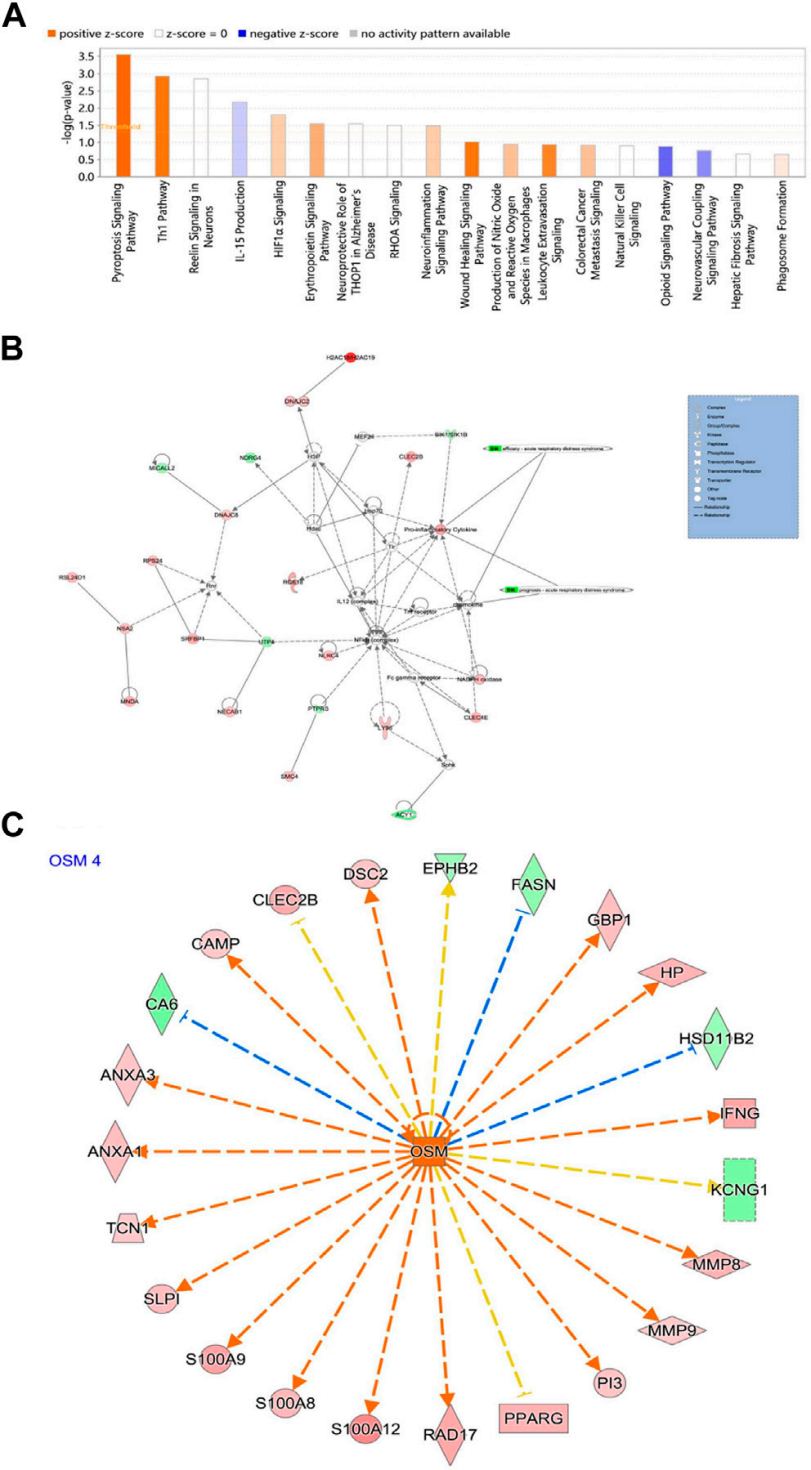
**(A)** Significant classical pathway enrichment results; **(B)** IPA interaction network; **(C)** Regulation of activated OSM. Red represents upregulated genes, green represents downregulated genes, orange arrows represent the process of activation, blue represents the process of inhibition, and orange represents inconsistency with the results of the study.

Classical pathway analysis showed that the 46 key genes were related to sepsis signal pathway, cellular immune response, HIF-1 signal pathway, erythropoietin triggering signal pathway in endothelial cells, neuroinflammation pathway, wound healing signal pathway, leukocyte overflow signal activation, IL-15 signal pathway, opioid signal pathway, inhibition of neurovascular coupling signal pathway, etc. (Figure 7A; Supplementary Table S3). Moreover, seven genes (APH1A, GAD1, GRINA, IFNG, KCNJ9, MMP9, and NCF1) were enriched in the Neuroinflammation Signaling Pathway. Genes IFNG and MMP9 were associated with Airway Inflammation in Asthma. The molecular interaction network is shown in Figure 7B. The upstream transcription factors were predicted and their action mode was determined. The results showed that most of the upregulated genes were activated, and most of the downregulated genes were inhibited (Figure 7C).

### Key Gene-Drug Interaction Network

**FIGURE 8:**
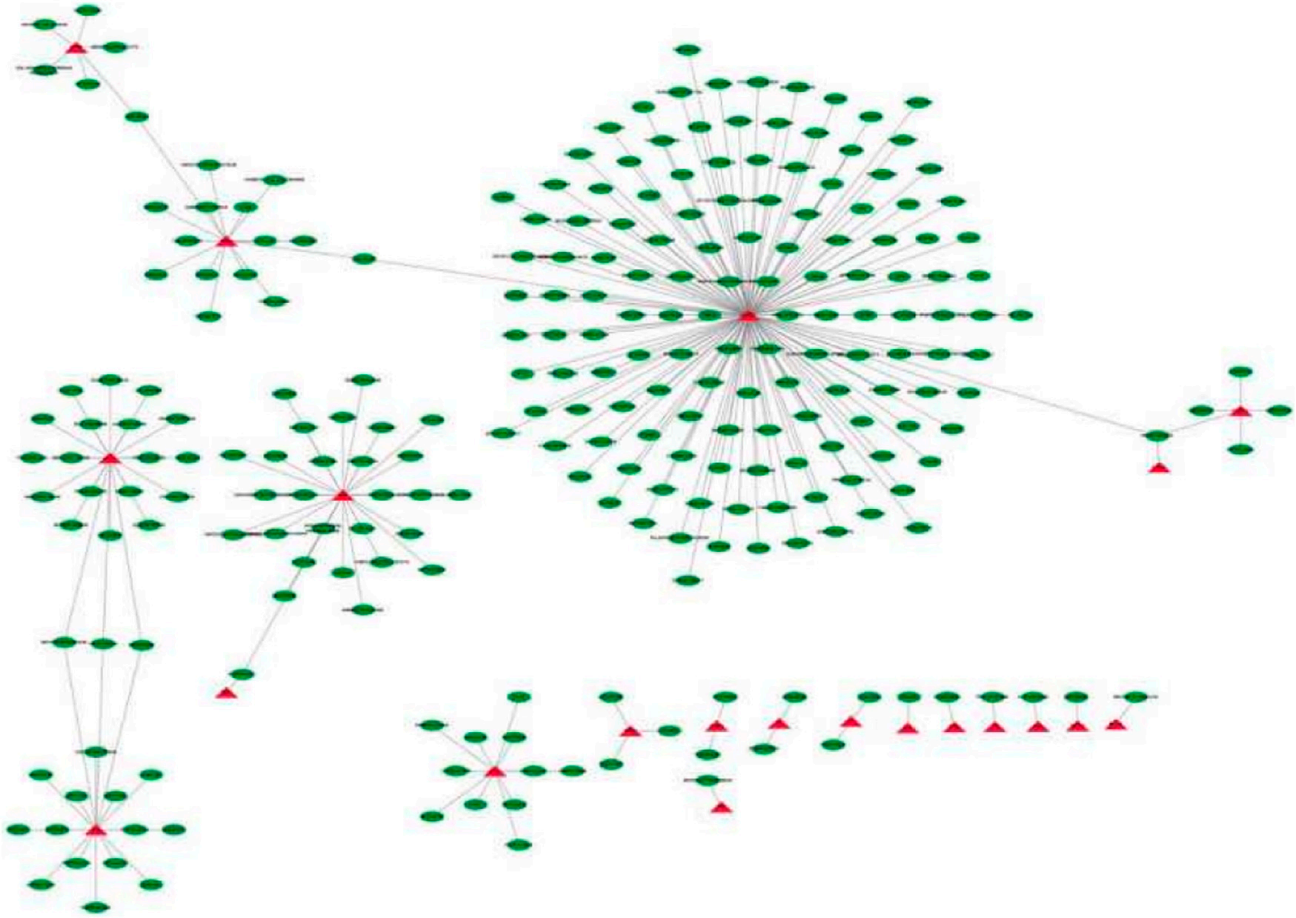
Core gene-drug interaction network The red triangle is the core gene and the green ellipse is the drug molecule.

In order to explore the potential therapeutic drugs for the diseases related to 46 key genes, the compounds targeting the proteins encoded by key genes were identified. There were 251 drugs with therapeutic effects on 21 key genes (Figure 8), including peroxisome proliferator-activated receptor *γ* (PPAR *γ*), hemoglobin-β-track (HBB), annexin 1 (ANXA1), interferon (IFNG), and matrix metalloproteinase.

## Discussion

In recent years, the respiratory and critical care medicine, respiratory treatment technology, and lung transplantation and other operations have been rapidly developed and improved, resulting in an increased incidence of benign tracheal stenosis (Chen et al., 2021). Although bronchoscopic intervention is a standard treatment for benign tracheal stenosis, it can cause secondary damage to the airway wall structure in some patients, and the excessive granulation tissue and scar contracture formed during the repair process will cause airway obstruction and force patients to undergo intervention therapy again, resulting in a vicious circle of “stenosis-treatment-repair-restenosis” (Jeong et al., 2020). The management of airway stenosis has become a challenge in the field of respiratory interventional therapy. Therefore, it is of great significance to explore the pathogenesis of benign tracheal stenosis and use effective methods to control its development.

In this study, by comparing tracheal stenosis samples with normal control samples, 229 differentially expressed mRNA, 40 differentially expressed lncRNA and 583 differentially expressed circRNA were identified, suggesting that these genes may be involved in the pathogenesis of benign tracheal stenosis. So far, there are few studies on lncRNA and circRNA in benign airway stenosis, and their functions need to be explored. Functional enrichment analysis showed that the differential mRNAs were significantly enriched in biological processes such as neutrophil degranulation, neutrophil activation involved in immune response, neutrophil mediated immunity, and neutrophil activation. It is speculated that the development of benign airway stenosis is related to the activation of neutrophils. It has been found that neutrophils can gather to the wound site within 24 h after injury (Welshhans and Hom, 2017), release inflammatory mediators and cytokines such as TNF- α, IL-1 and IL-6, enhance inflammatory response, and stimulate vascular endothelial growth factor and IL-8 production, which all serve to facilitate injury repair (Reinke and Sorg, 2012). However, neutrophils also produce a large amount of oxygen free radicals, which can cause oxidative stress in wound tissue, delay the repair process and change the healing result (Koh and DiPietro, 2011). It has been reported that after the operation on New Zealand rabbits, the tissue wound was accompanied by a large number of inflammatory cell infiltration such as neutrophils and macrophages (Zhang et al., 2021).

Protein-protein interaction network is a network with proteins as nodes, and the edges indicates that the proteins participate in the same metabolic pathway, biological process, structural complex, functional correlation, or have physical contact. Proteins interact with each other to regulate various life processes such as biological signal transduction, gene expression regulation, energy and substance metabolism, and cell cycle (Wang et al., 2014). In this study, the PPI network of 229 differential mRNAs was constructed, and the interaction relationships of 160 proteins was obtained. The upregulated genes such as matrix metalloproteinase (MMP9), calprotectin S100A9/12/8, serum haptoglobin (HP) and camp were located in the center of the network, suggesting that these genes were involved in disease development. 46 key genes were obtained by further screening. The key genes were significantly related to cellular immune response, HIF-1 signaling pathway, erythropoietin triggering signaling pathway in endothelial cells, neuroinflammatory pathway, wound healing signaling pathway and interleukin regulation. It has been found that IL-8 can not only promote the gap junction communication between fibroblasts, accelerate the maturation of granulation tissue (Moyer et al., 2002), but also promote the proliferation of fibroblasts and change the morphology of fibroblasts (Lim et al., 2009). As a chemokine, IL-8 can participate in the inflammatory response, induce the aggregation of polymorphonuclear leukocytes (Lira and Furtado, 2012; Puyo and Dahms, 2012), and participate in the activation of leukocytes in the wound healing process (Mukaida et al., 1998). Through co-expression network analysis and construction of ce-RNA network, a ce-RNA network of “lnc-RNA- miRNA-mRNA” composed of 8 lnc-RNAs, 31 mRNAs and 116 miRNAs, and a ce-RNA network of “circRNA- miRNA-mRNA” composed of 30 circRNAs, 36 mRNAs and 275 miRNAs were obtained. Some drugs have been found to inhibit cell cycle progression and promote the apoptosis of primary human airway granulation fibroblasts through Mir143Hg (LNCRNA)/Mir-1275 (Mirna)/ILK axis, thus effectively inhibiting the proliferation of airway granulation tissue and ultimately alleviating airway stenosis, which is a potential therapy for benign airway stenosis (Zhang et al., 2021). This study can provide more theoretical support for similar regulatory pathways and therapeutic targets.

Hemoglobin A2(HbA2), calcium binding protein S100A, matrix metalloproteinase (MMP) and growth differentiation factor-related serum protein (WFIKKN1) are the key genes identified in this study, and they are important nodes in each relationship network, suggesting that these genes play an important role in the pathogenesis of benign airway stenosis. Patients with benign airway stenosis are often accompanied by edema and congestion of airway mucosa in pharynx, or clinical symptoms such as congestion, bleeding, erosion or scar formation of trachea and bronchial mucosa; moreover, hemoglobin A2 level is often increased in these patients (Takaishi et al., 2021). Therefore, the abnormal level of hemoglobin A2 is one of the important features of benign airway stenosis. Calcium binding protein S100A participates in innate immunity and plays a role in recruiting leukocytes and neutrophils in inflammatory tissues (Tong et al., 2014). The first stage of airway injury repair is inflammatory response: neutrophils, macrophages and other inflammatory cells infiltrate and release chemokines. It has been proposed that calcium binding protein S100A may be involved in the repair of airway injury. In addition, calcium binding protein S100A interacts with extracellular matrix proteins to stimulate fibroblast proliferation and regulate leukocyte metastasis (Zhong et al., 2016). During the repair process of airway injury, granulation tissue is fibrosed, new epithelium is formed, matrix is remodeled, and the repair of injury is completed (Stunova and Vistejnova, 2018). Low expression of lncRNA LINC00665 inhibits the invasion and proliferation of bladder cancer by regulating S100A13. It has been found that S100A9 is involved in idiopathic non-fibrotic process and can be used as an index for disease evaluation and monitoring (Korthagen et al., 2010). The expression of S100A9 was significantly upregulated in our study, but whether it functioned as previously reported needs further investigation. It has been shown that matrix metalloproteinase MMP9 can effectively degrade the main components of extracellular matrix and play an important role in the process of cancer cell abscission, invasion and metastasis. The activation of MMP-9 is responsible for the degradation of tight junction protein and the opening of tight junction, resulting in increased endothelial cell permeability (Liu et al., 2018). Sevoflurane treatment can relieve neuron damage by inhibiting the expression of MMP-9 in microglia after spinal cord ischemia-reperfusion injury in rats (Li et al., 2014). In our study, the expression of MMP9 was up-regulated, and its protein level needs to be measured to explore its function. Growth factor-related serum protein (WFIKKN1) is a myostatin and growth differentiation factor inhibitor related to muscle growth (Szláma et al., 2013). The WFIKKN expression is increased in human serum during resistance training, as well as during the growth of rat longitudinal muscle (Aoki et al., 2009). In our study, WFIKKN1 expression decreased in benign airway stenosis, which is opposite to what we expected; thus, its protein level needs to be measured and its functions need to be explored in future studies.

In animal cells, HIF (hypoxia-inducible factor) and its regulator pVHL (VonHippel-Lindau tumor suppressor protein) were involved in the perception and response of oxygen. In the presence of hypoxia, the HIF-1α subunit could not be recognized by pVHL. The dimer was formed and transferred to the nucleus, interacted with the cofactor CBP/p300 and Pol II complex, and bound with HRE (hypoxia response element) to activate the transcription of target genes. In addition, airway stenosis was closely related to mechanical compression. Under the compression of the air bag, the tracheal mucosa was ischemia and hypoxia, and the synthesis of hypoxia-inducible factor (HIF-1α) promoted the overexpression of TGF-β and VEGF. Fibroblasts activated and proliferated excessively, which resulted in airway scarring (Cai et al., 2013).

At present, the treatment of benign airway stenosis mainly includes surgery, intervention therapy, and drug therapy. In this study, the potential therapeutic drugs targeting the 46 key genes were analyzed, the results showed that the key gene PPAR *γ* had the most targeting drugs, indicating that this gene may play an important role in the development of benign airway stenosis. PPARs belonged to the nuclear hormone receptor superfamily and it consisted of three members, PPAR-α, PPAR-β/δ, and PPAR-γ (Han et al., 2017). The function of PPARs was mainly through the interaction of ligands with co-activators or co-repressors (Brunmeir and Xu, 2018). Carvalho et al. (2021). found that PPAR γ suppressed the gene expression of inflammatory mediators by trans-repressing other transcription factors, thereby controlling the exacerbated inflammation that occured in lung injury/acute respiratory distress. In recent years, with the gradual deepening of the research on PPAR-γ, it had been found that PPAR-γ played an important role in the pathogenesis of chronic respiratory diseases, such as asthma, chronic obstructive pulmonary disease (COPD), lung cancer and pulmonary fibrosis. The application of PPAR-γ agonists was expected to become a new method for the treatment of chronic diseases of the respiratory system (Zhang et al., 2019; Pan et al., 2021; Xu et al., 2021). It has been proposed that PPAR *γ* may be responsible for initiating the endogenous mechanism of wound repair and controlling fibrotic response by activating PPAR *γ* through its natural ligand (Wu et al., 2009; Wei et al., 2010). Therefore, PPAR *γ* may be a potential therapeutic target for treating benign airway stenosis.

In this study, the expression of benign airway stenosis and control transcripts was rapidly obtained by whole transcriptome sequencing. Compared with conventional transcriptome sequencing, whole transcriptome sequencing can simultaneously obtain three types of RNA information: mRNA, lncRNA and circRNA. So far, no whole transcriptome sequencing articles related to this disease have been consulted. The innovation of this study was sequenced using its own samples, and to explore potential therapeutic drugs for key gene-related diseases. This study constructed a ceRNA network through bioinformatics methods, analyzed the main functions of key genes, and predicted potential therapeutic drugs, which provided a certain reference for the pathogenesis of benign tracheal stenosis, but this study lacked *in vitro* and *in vivo* experimental verification. We would further explore its mechanism of action and verify the function of key genes in follow-up clinical experiments.

In conclusion, our study analyzed the differential mRNA, lncRNA and circRNA between patients with benign airway stenosis and normal controls. Two ceRNA networks were constructed, which provide a new perspective for further exploring the pathogenesis of benign airway stenosis. In addition, the discovery of potential drugs for treating benign airway stenosis, opening up a new direction for the drug development of this disease.

## Data Availability

The original contributions presented in the study are publicly available. This data can be found here: https://www.ncbi.nlm.nih.gov/geo/query/acc.cgi?acc=GSE206250.
