# Supplementary figures and images for: Construction and Comprehensive Analysis of the ceRNA Network to Reveal Key Genes for Benign Tracheal Stenosis
Source: Front Genet. 2022 Jun 23;13:891741. doi: 10.3389/fgene.2022.891741 (PMC9261475; doi:10.3389/fgene.2022.891741)

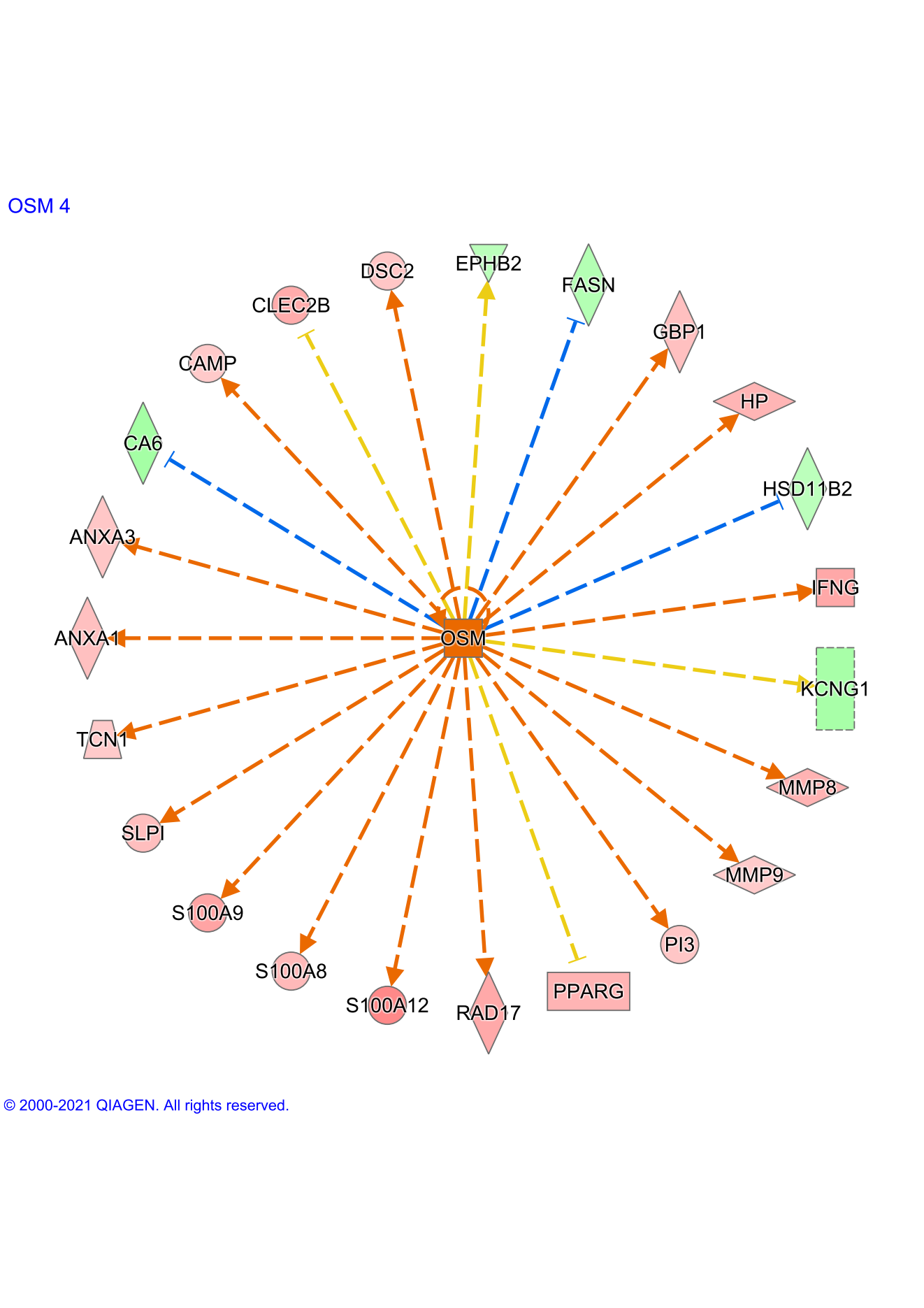

Supplement: Supplementary file 3 [file Image6.TIF]

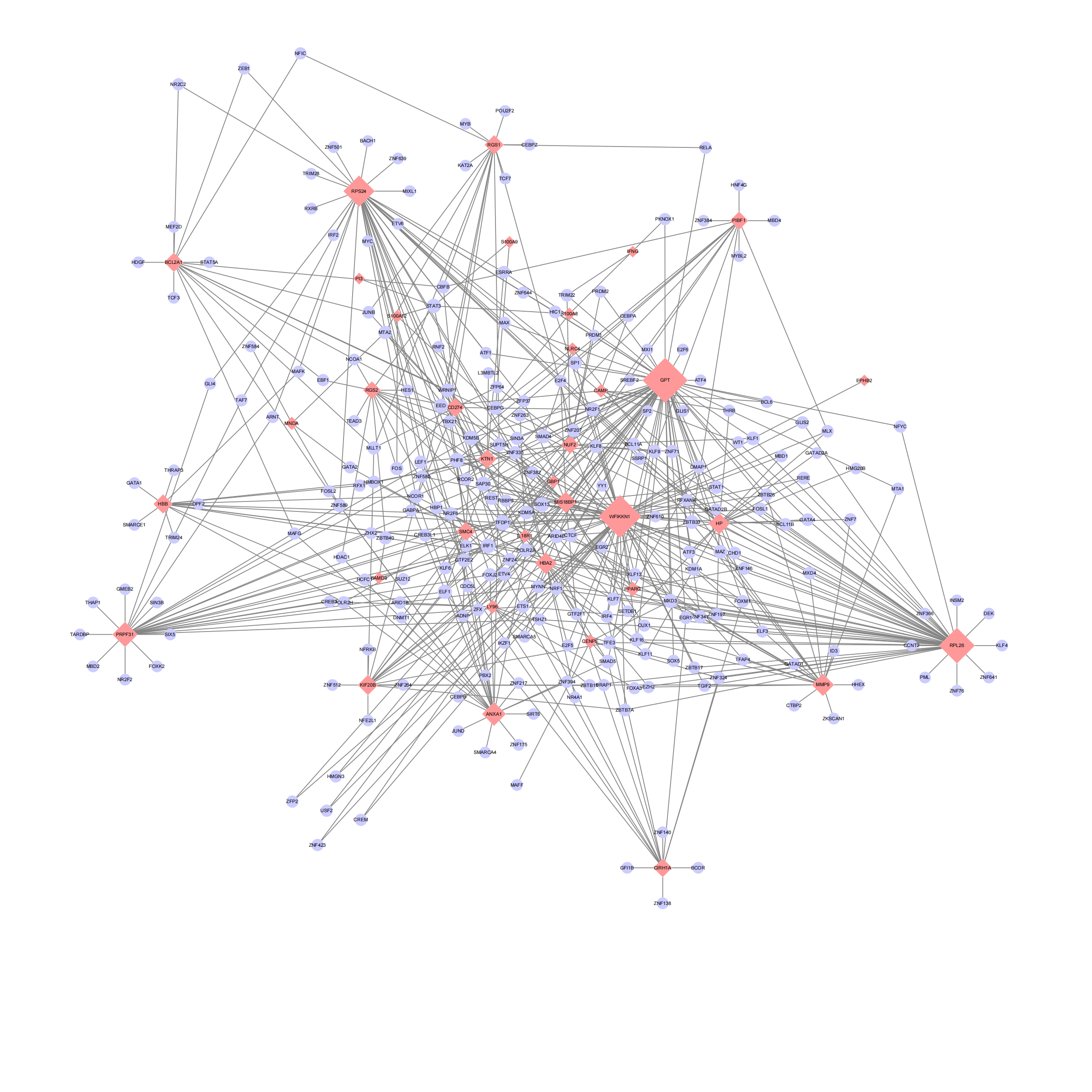

Supplement: Supplementary file 4 [file Image3.TIF]

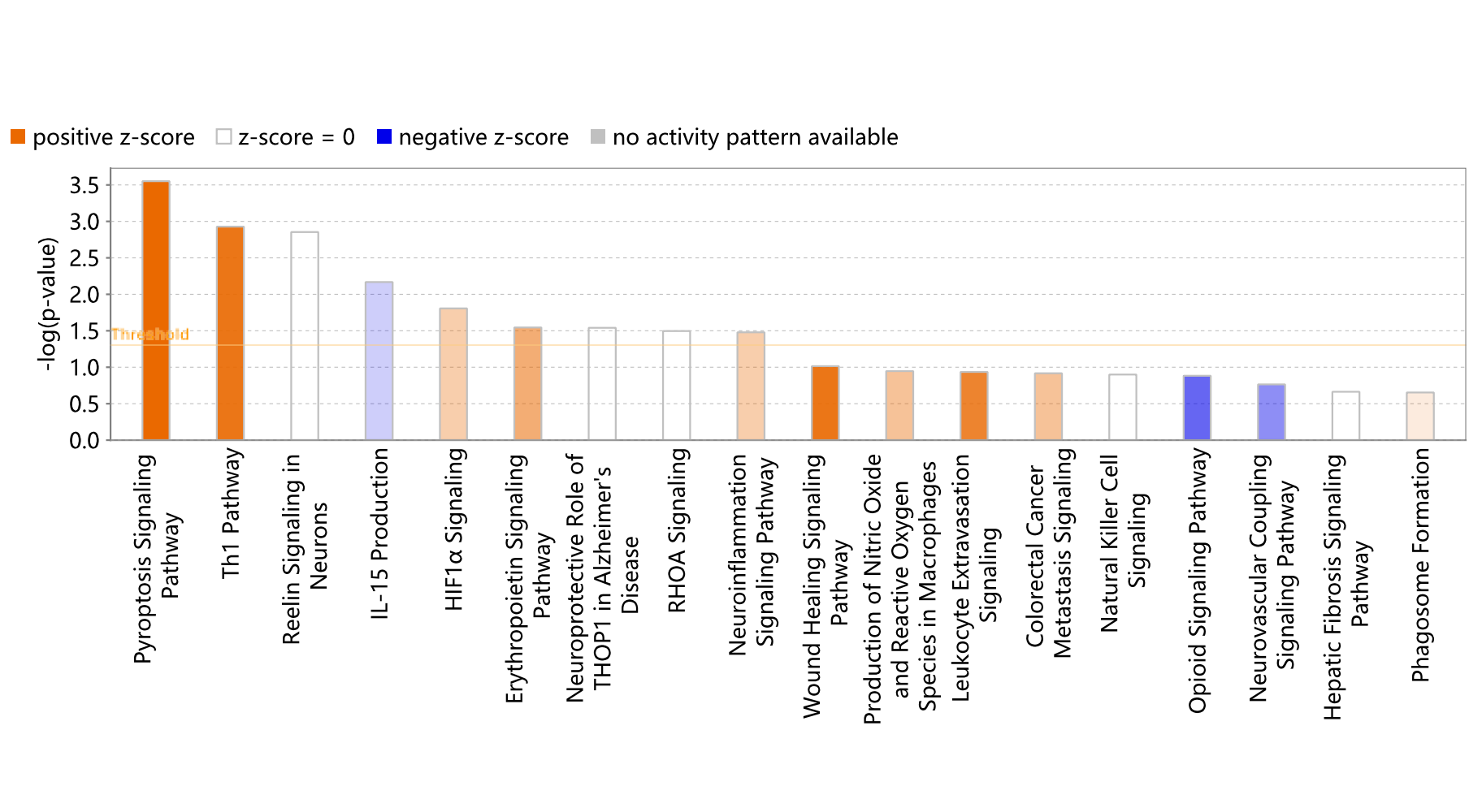

Supplement: Supplementary file 5 [file Image4.TIF]

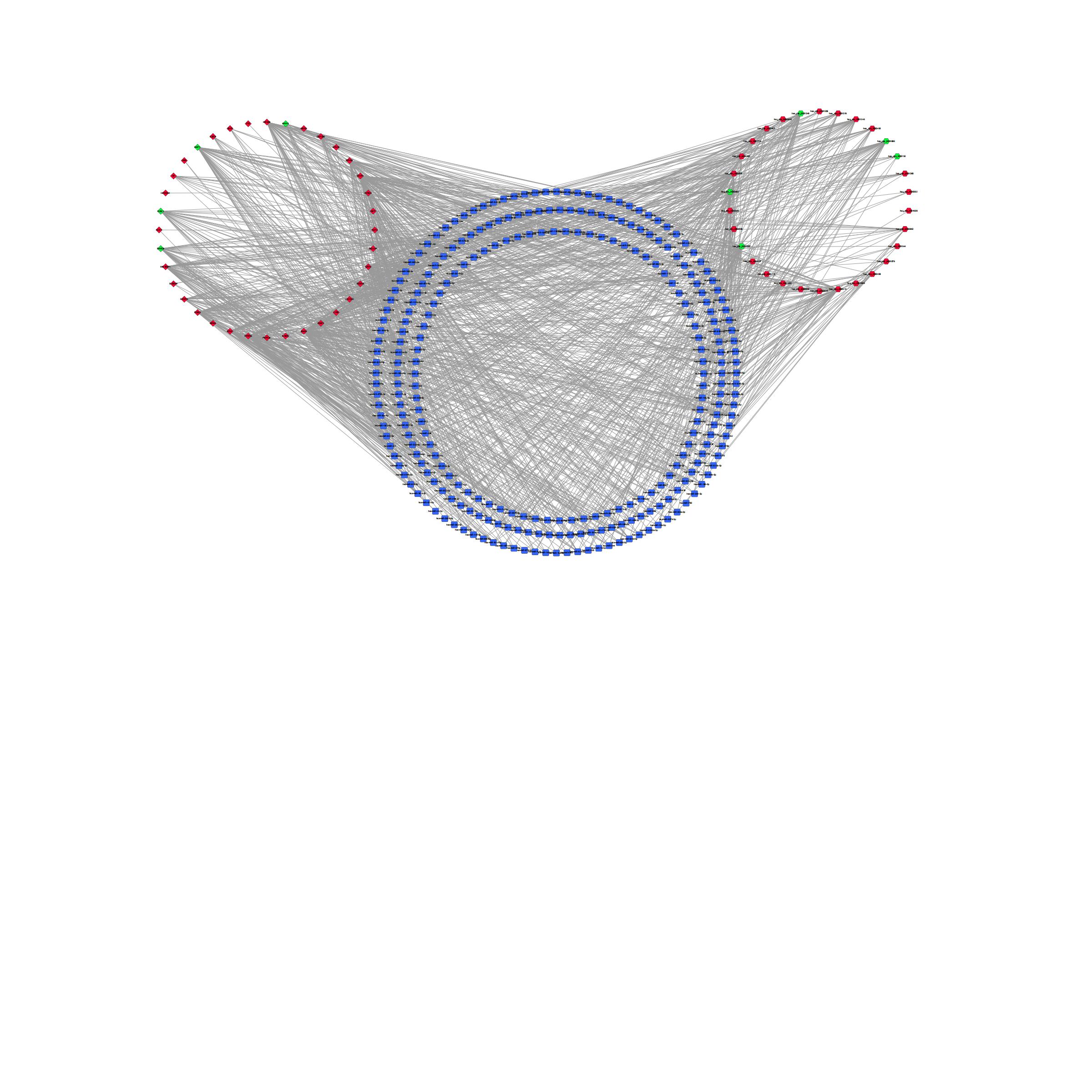

Supplement: Supplementary file 6 [file Image1.TIF]

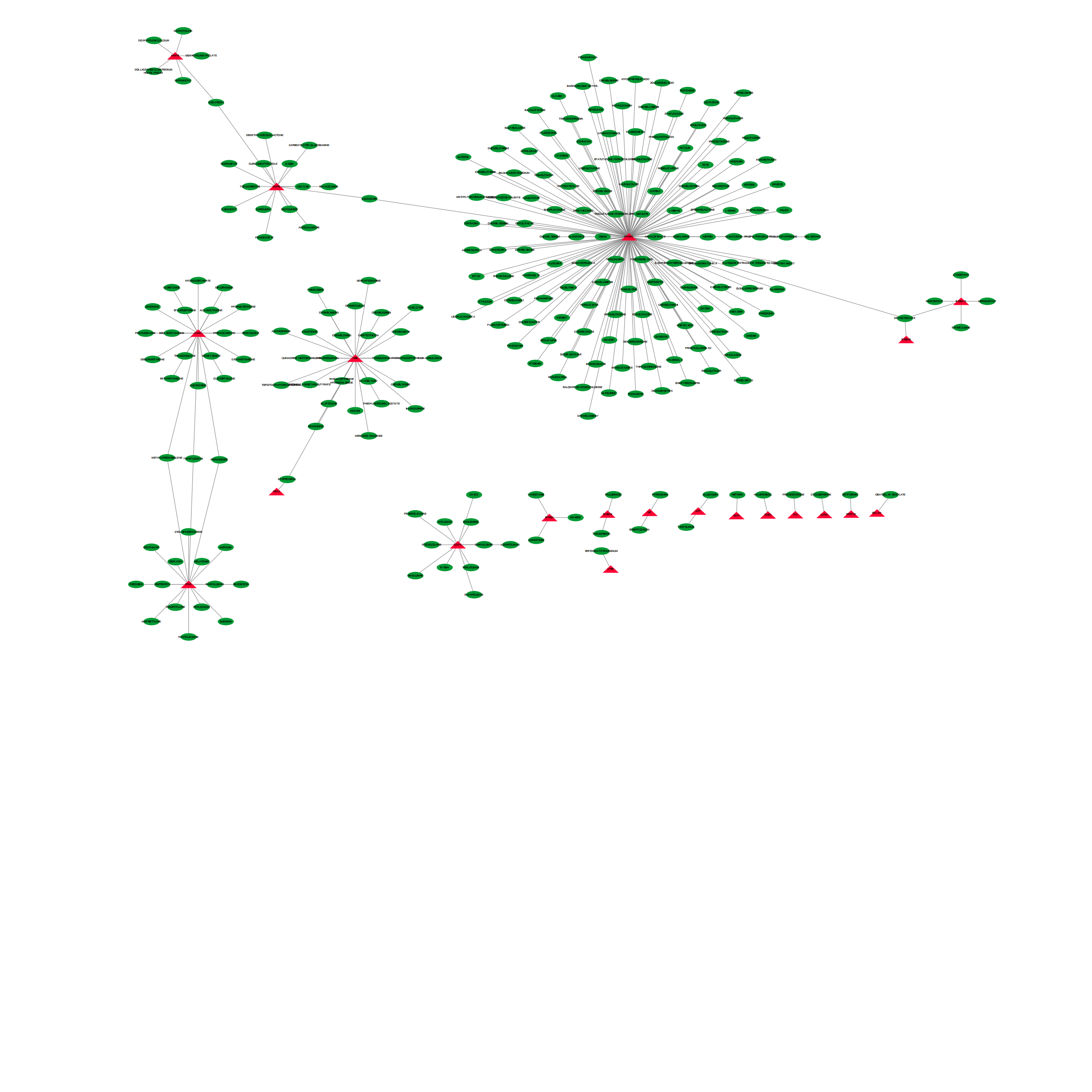

Supplement: Supplementary file 7 [file Image7.TIF]

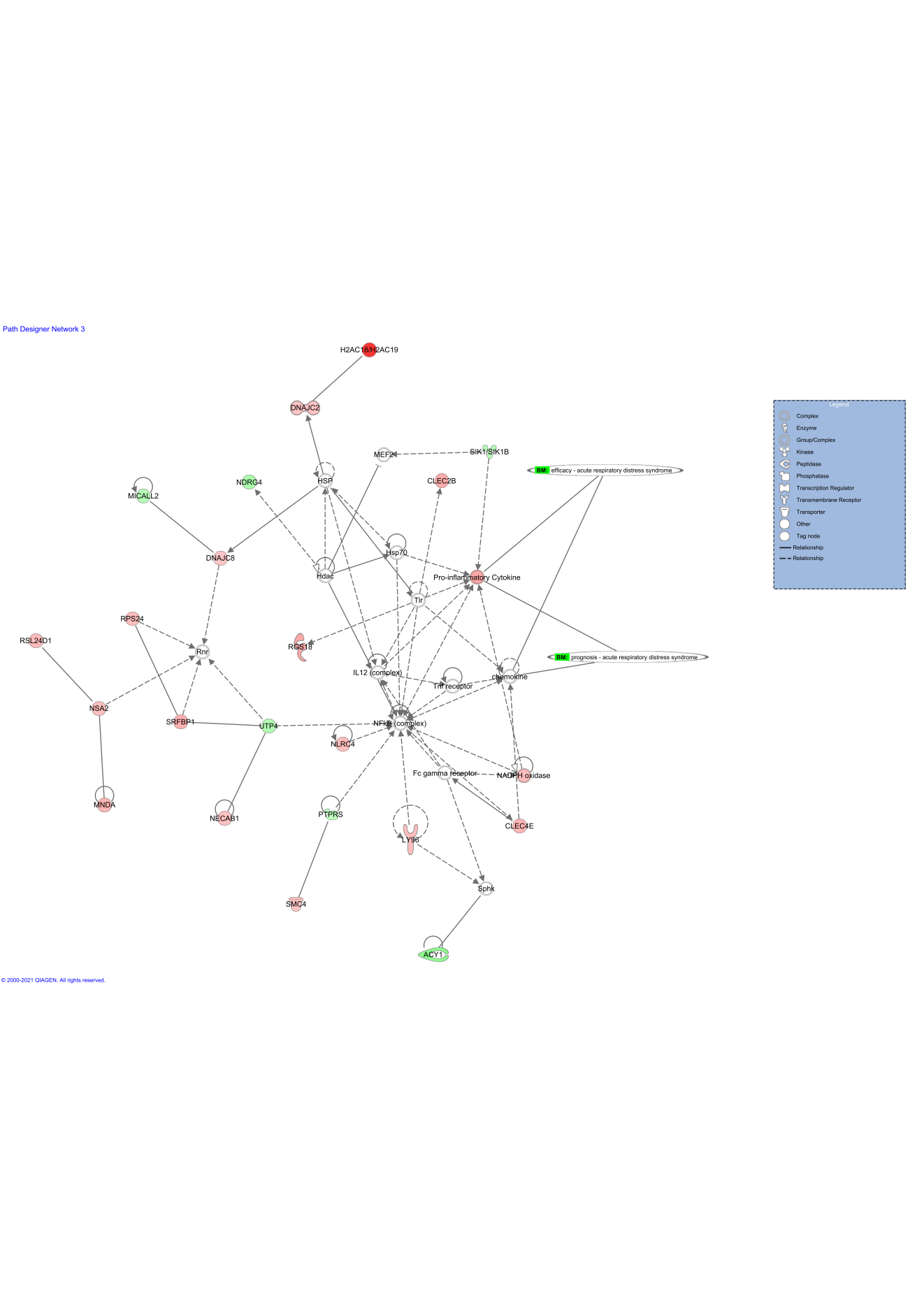

Supplement: Supplementary file 9 [file Image5.TIF]

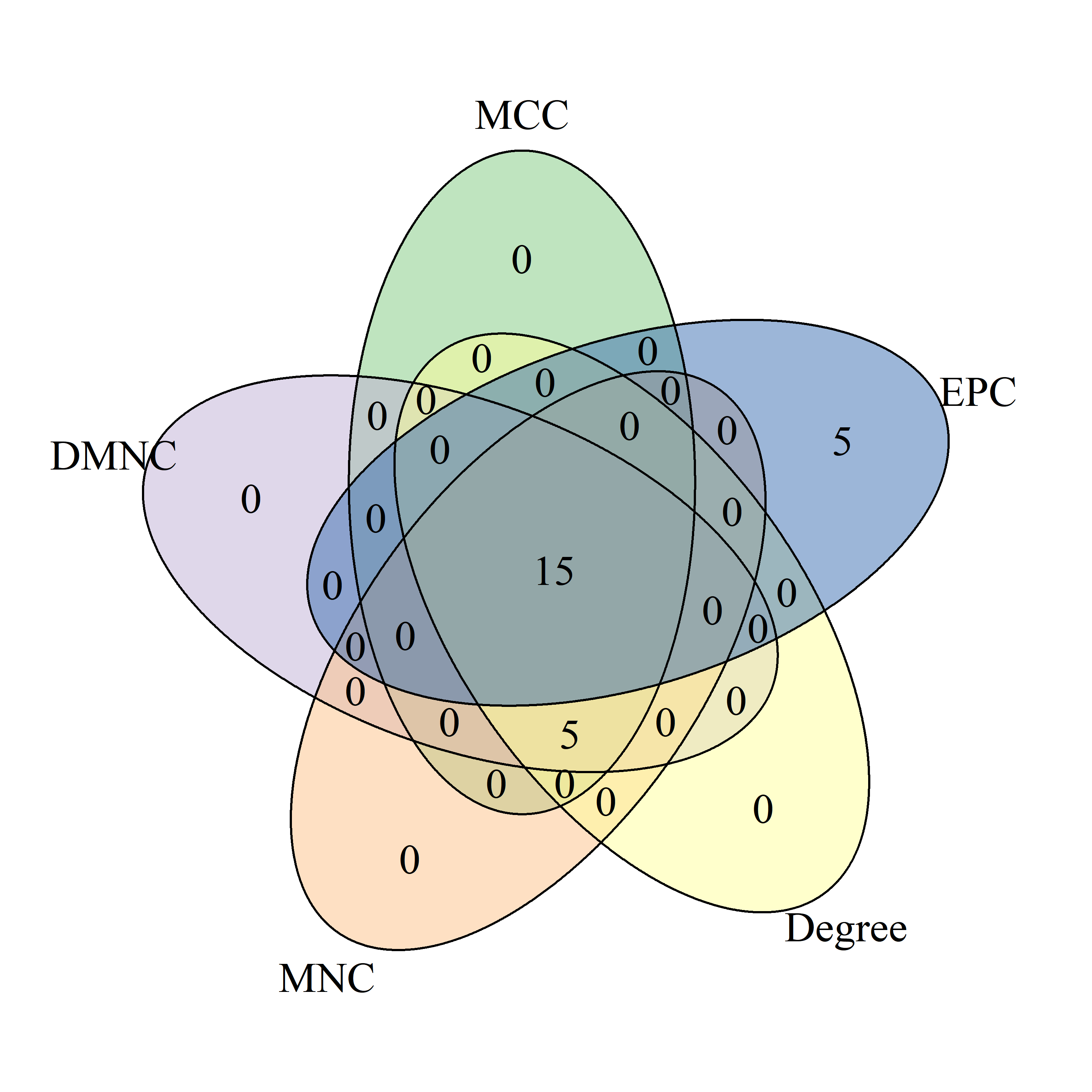

Supplement: Supplementary file 10 [file Image2.TIFF]
